# histoneHMM: Differential analysis of histone modifications with broad genomic footprints

**DOI:** 10.1186/s12859-015-0491-6

**Published:** 2015-02-22

**Authors:** Matthias Heinig, Maria Colomé-Tatché, Aaron Taudt, Carola Rintisch, Sebastian Schafer, Michal Pravenec, Norbert Hubner, Martin Vingron, Frank Johannes

**Affiliations:** 1 Department of Computational Molecular Biology, Max Planck Institute for Molecular Genetics, Ihnesstrasse 63-73, Berlin, 14195 Germany; 2 Experimental Genetics Group, Max-Delbrück-Center for Molecular Medicine, Robert-Rössle-Strasse 10, 13092Berlin, Germany; 3 Quantitative Epigenetics, European Research Institute for the Biology of Ageing, University of Groningen, University Medical Center Groningen, A. Deusinglaan 1, AV, Groningen, 9713 The Netherlands; 4 Institute of Physiology, Academy of Sciences of the Czeck Republic, Videnska 1083, Prague, 14220 Czech Republic; 5 Groningen Bioinformatics Center, University of Groningen, Nijenborgh 7, AG, Groningen, 9747 The Netherlands

**Keywords:** ChIP-seq, Histone modifications, Hidden Markov model, Computational biology, Differential analysis

## Abstract

**Background:**

ChIP-seq has become a routine method for interrogating the genome-wide distribution of various histone modifications. An important experimental goal is to compare the ChIP-seq profiles between an experimental sample and a reference sample, and to identify regions that show differential enrichment. However, comparative analysis of samples remains challenging for histone modifications with broad domains, such as heterochromatin-associated H3K27me3, as most ChIP-seq algorithms are designed to detect well defined peak-like features.

**Results:**

To address this limitation we introduce histoneHMM, a powerful bivariate Hidden Markov Model for the differential analysis of histone modifications with broad genomic footprints. histoneHMM aggregates short-reads over larger regions and takes the resulting bivariate read counts as inputs for an unsupervised classification procedure, requiring no further tuning parameters. histoneHMM outputs probabilistic classifications of genomic regions as being either modified in both samples, unmodified in both samples or differentially modified between samples. We extensively tested histoneHMM in the context of two broad repressive marks, H3K27me3 and H3K9me3, and evaluated region calls with follow up qPCR as well as RNA-seq data. Our results show that histoneHMM outperforms competing methods in detecting functionally relevant differentially modified regions.

**Conclusion:**

histoneHMM is a fast algorithm written in C++ and compiled as an R package. It runs in the popular R computing environment and thus seamlessly integrates with the extensive bioinformatic tool sets available through Bioconductor. This makeshistoneHMM an attractive choice for the differential analysis of ChIP-seq data. Software is available from http://histonehmm.molgen.mpg.de.

**Electronic supplementary material:**

The online version of this article (doi:10.1186/s12859-015-0491-6) contains supplementary material, which is available to authorized users.

## Background

Post-translational modifications of histones, such as methylation, acetylation, phosphorylation or ubiquitination have central roles in genome function [1] and in the preservation of genome integrity [2]. These epigenetic marks participate in the silencing of transposable elements [3] as well as in the regulation of specific genes during development [4]. Improper placement of histone modifications can lead to abnormal cellular phenotypes such as those observed in cancers [5], during aging [6], or in response to certain environmental/nutritional challenges [7].

Genome-wide measurements of various histone modifications can be readily obtained using ChIP-seq technologies, which combine immmunoprecipation techniques with next generation sequencing [8]. Although the application of this technology has become routine in most laboratories, downstream computational analyses continue to be a major bottleneck for many experimentalists. A common experimental goal is to compare the ChIP-seq profiles between an experimental sample (e.g. cancer sample) and a reference sample (e.g. normal controls), and to identify regions that show differential modification patterns. These regions can be used to identify genes and regulatory mechanisms involved in diverse biological processes such as development or disease.

**Figure 1:**
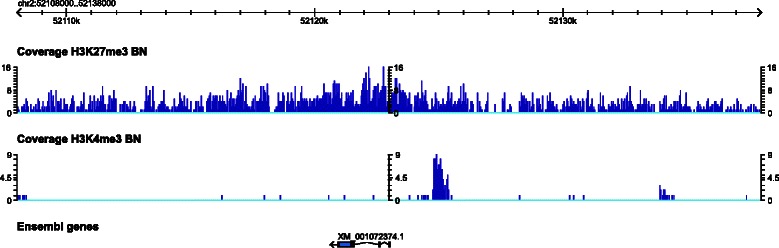
**Example genome browser screen-shot.** ChIP-seq read coverage of H3K27me3 (upper coverage track) occurs in broad domains across the genome compared to other histone marks like H3K4me3 (lower coverage track), which occur in precisely defined peaks. Data from [15].

Several methods have been developed to facilitate comparisons of ChIP-seq samples for peak-like features [9,10]. However, many important histone modifications do not occur in narrow well-defined peaks, but show broad diffuse patterns (Figure 1). H3K27me3, for example, is a histone modification that is deposited by the polycomb group of proteins [1]. Together with H3K9 methylation, it forms large heterochromatic domains [11] which can span several thousands of basepairs [12,13]. Even with deeply sequenced ChIP-seq libraries, histone modifications of this type can yield relatively low read coverage in effectively modified regions, thus producing low signal to noise ratios. Application of methods that search for peak-like features in such data can generate many false positive or false negative calls. These miscalls compromise downstream biological interpretations and affect decisions regarding experimental follow-up studies.

To address these issues we developed histoneHMM, a novel bivariate Hidden Markov Model for the differential analysis of histone modifications with broad genomic footprints. histoneHMM aggregates short-reads over larger regions and takes the resulting bivariate read counts as inputs for an unsupervised classification procedure, requiring no further tuning parameters. histoneHMM outputs probabilistic classifications of genomic regions as being either modified in both samples, unmodified in both samples or differentially modified between samples.

We extensively evaluate the performance of histoneHMM in the context of ChIP-seq data of two broad repressive histone marks, H3K27me3 and H3K9me3 from rat, mouse and human cell lines. Using several biological criteria and follow-up experimental validation, we show that histoneHMM outperforms competing methods in calling differentially modified regions between samples.

histoneHMM is a fast algorithm written in C++ and compiled as an R package. It runs in the popular R computing environment and thus seamlessly integrates with the extensive bioinformatic tool sets available through Bioconductor. This makes histoneHMM an attractive choice for the differential analysis of ChIP-seq data.

## Results and discussion

### Genome-wide detection of differentially modified regions

We analyzed ChIP-seq data collected from the left ventricle of the heart of two different inbred rat strains, Spontaneously Hypertensive Rat (SHR/Ola) and Brown Norway (BN-Lx/Cub). SHR is a classical animal model for hypertension which is extensively used in studies of cardiovascular disease[14]. The biological motivation was to compare the heart epigenomes of these two strains in order to identify candidate regions that contribute to the hypertensive phenotype in SHR. Here we focused on data for the repressive mark H3K27me3, which was generated as part of a larger study to characterize the impact of sequence variation on histone marks in the rat [15]. Further, we extended our analysis to H3K9me3, another repressive histone mark. This second data set was previously used to study sex specific histone marks in the liver of CD-1 mice [16]. Finally, we analyzed the differential enrichment of H3K27me3, H3K9me3, H3K36me3 and H3K79me2 between the human embryonic stem cell line H1-hESC (H1) and the K562 cell line, using data provided by the ENCODE project [17].

All of the analyzed histone marks and especially H3K27me3 and H3K9m3 are known to have large genomic footprints that can extend up to several thousands basepairs in length [12,13]. To evaluate the performance of histoneHMM, we applied four competing algorithms to these data, Diffreps [18], Chipdiff [19], Pepr [20] and Rseg [21]. Similar to histoneHMM, these algorithms are designed for the differential analysis of ChIP-seq experiments, and are not restricted to narrow peak-like data, thus providing a suitable reference. Biological replicates were available for all of the modifications (Table 1). The reads from all strain replicates were merged and used for analysis. Following other methods [18,19], we binned the genome into 1000 bp windows, and aggregated read counts within each window.

**Table 1:** **Overview of ChIP-seq and RNA-seq sequencing data for the rat (BN and SHR), for the mouse (male and female), for the myoblast (MB) dataset from [** 36 **], and for the ENCODE cell lines**

**Data**	**Replicate**	**Total number**	**Mapped**
		**of reads**	**reads**
H3K27me3 BN	1	69,047,384	54,415,680
H3K27me3 BN	2	82,631,022	70,349,074
H3K27me3 BN	3	70,920,377	60,263,098
H3K27me3 SHR	1	62,894,736	49,966,171
H3K27me3 SHR	2	68,439,111	58,495,532
H3K27me3 SHR	3	68,419,655	58,433,557
Input BN	1	14,104,386	12,833,263
Input BN	2	15,381,807	14,172,254
Input BN	3	61,276,324	58,969,661
Input SHR	1	16,049,419	14,700,053
Input SHR	2	18,586,414	16,910,655
Input SHR	3	74,035,329	70,234,582
RNA-seq BN	1	168,796,774	121,417,255
RNA-seq BN	2	162,380,800	123,606,276
RNA-seq BN	3	170,100,328	129,078,242
RNA-seq BN	4	161,444,260	117,512,826
RNA-seq BN	5	144,182,176	105,095,363
RNA-seq SHR	1	164,552,150	118,500,162
RNA-seq SHR	2	166,005,952	126,920,455
RNA-seq SHR	3	149,525,162	108,870,073
RNA-seq SHR	4	120,844,554	85,337,460
RNA-seq SHR	5	138,124,004	99,015,155
H3K9me3 female	1	13,306,841	8,708,647
H3K9me3 female	2	7,860,660	3,942,578
H3K9me3 female	3	7,092,019	3,553,617
H3K9me3 male	1	12,091,621	6,274,126
H3K9me3 male	2	7,195,641	3,514,546
H3K9me3 male	3	5,703,768	2,786,109
Input	1	10,458,196	6,714,959
Input	2	4,304,875	1,940,625
Input	3	3,586,754	1,559,414
Input	4	4,482,286	1,947,833
Input	5	3,680,479	1,535,794
Input	6	14,922,773	11,291,654
RNA-seq female	1	6,245,431	4,367,593
RNA-seq female	2	13,093,629	9,257,763
RNA-seq male	1	14,086,627	11,311,584
RNA-seq male	2	8,098,083	6,476,421
MB H3K27me3	1	9,453,468	9,421,380
MB H3K27me3	2	9,924,308	9,875,240
MB H3K27me3	3	10,316,946	10,262,585
MB input	1	9,798,009	9,780,084
MB input	2	9,807,040	9,789,125
MB input	3	7,351,896	7,340,980
MB input	4	12,350,738	12,324,079
H1 H3K09me3	1	32,382,686	22,900,208
H1 H3K09me3	2	41,645,083	27,715,874
H1 H3K27me3	1	8,342,672	6,434,801
H1 H3K27me3	2	15,963,714	12,146,581
H1 H3K27me3	3	19,825,041	10,943,029
H1 H3K27me3	4	17,600,144	5,009,090
H1 H3K27me3	5	11,715,209	7,194,836
H1 H3K27me3	6	14,948,211	8,705,091
H1 H3K27me3	7	13,277,331	5,472,352
H1 H3K36me3	1	24,086,746	13,669,344
H1 H3K36me3	2	16,739,261	13,164,807
H1 H3K79me2	1	29,740,715	24,616,670
H1 H3K79me2	2	45,788,899	35,599,680
H1 input	1	13,876,810	10,813,095
H1 input	2	16,581,567	7,681,001
H1 RNA-seq	1	250,790,392	140,719,829
H1 RNA-seq	2	214,202,680	114,403,031
K562 H3K27me3	1	19,297,190	12,210,065
K562 H3K27me3	2	22,830,589	12,119,288
K562 H3K36me3	1	26,973,698	14,803,144
K562 H3K36me3	2	17,501,267	10,393,298
K562 H3K79me2	1	31,690,813	22,740,997
K562 H3K79me2	2	21,245,046	13,669,674
K562 H3K9me3	1	21,099,652	15,816,227
K562 H3K9me3	2	46,226,003	33,939,687
K562 input	1	27,579,809	19,570,350
K562 RNA-seq	1	227,177,516	134,666,953
K562 RNA-seq	2	238,106,630	158,075,847

Mapped reads refers to the number of uniquely mapped reads after removal of likely PCR duplicates.

**Figure 2:**
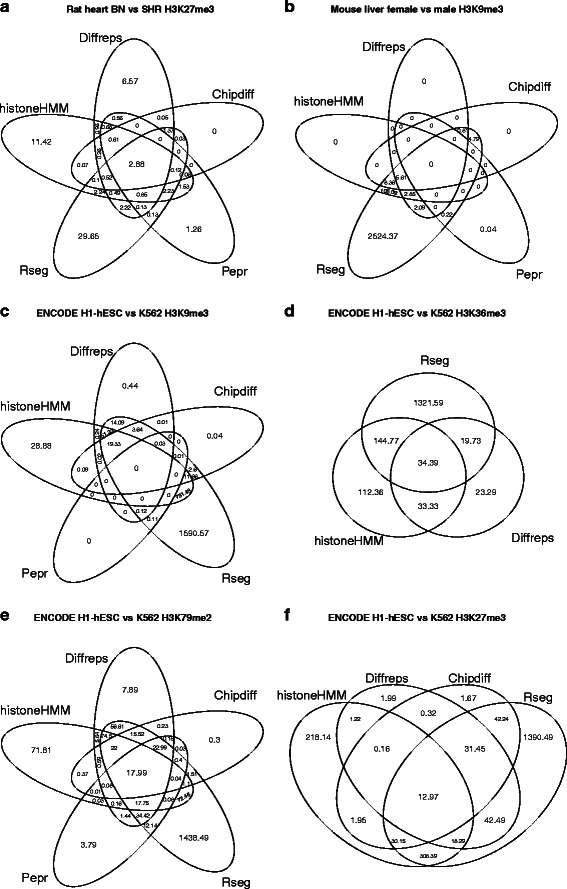
**Venn diagram.** The Venn diagrams show the overlap in Mb between the regions that were called differentially modified by each of the methods for the analysis of **a)** strain differences of H3K27me3, **b)** sex differences of H3K9me3 and differences between ENCODE cell lines H1-hESC and K562 for **c)** H3K9me3, **d)** H3K36me3 (Chipdiff and Pepr did not identify any differential regions), **e)** H3K79me2 and **f)** H3K27me3 (Pepr did not identify any differential regions).

Genome-wide, histoneHMM detected 24.96 Mb (0.9% of the rat genome) as being differentially modified between the two strains for H3K27me3, and 121.89 Mb as differentially modified between male and female mice for H3K9me3 (4.6% of the mouse genome) (Table 2). The analysis of ENCODE cell lines generally identified larger parts of the genome as differentially modified (9%-26% of the human genome) compared to the analysis of the same tissue between strains or sexes (Table 2). When comparing differential H3K27me3 and H3K9me3 regions, the number of regions reported by histoneHMM are greater than those reported by Diffreps and Chipdiff, however Rseg consistently detected an even larger number of modified regions. While a substantial part of the detected regions did overlap between methods (Figure 2), also a considerable proportion of regions were algorithm-specific. To explore the biological implications of this discrepancy we performed exemplary follow-up analyses for H3K27me3 and H3K9me3. For H3K27me3 we performed targeted qPCR on a selected number of regions for the SHR and BN strains, as well as RNA-seq expression experiments and functional annotation analysis. In addition we also explored the relation between differential H3K27me3 regions and differential binding of the polycomb complex in ENCODE cell lines. For H3K9me3 we studied X-inactivated genes as well as expression experiments. For the remaining ENCODE data sets, we evaluated the differential calls using gene expression data.

**Table 2:** **Detection of differentially modified regions (in Mb) between SHR and BN in the left ventricle of the heart for H3K27me3, between female and male mice for H3K9me3 and between H1-hESC and K562 ENCODE cell lines for H3K9me3, H3K79me2, H3K36me3 and H3K27me3**

	**histoneHMM**	**Diffreps**	**Chipdiff**	**Pepr**	**Rseg**
H3K27me3	24.96	18.09	6.08	11.05	42.97
H3K9me3	121.89	11.34	19.56	0.00	2651.86
ENCODE H3K9me3	843.03	89.26	37.43	0.27	2424.99
ENCODE H3K79me2	284.58	278.12	83.27	111.49	1788.00
ENCODE H3K36me3	324.85	110.74	0.00	0.00	1520.48
ENCODE H3K27me3	591.27	108.88	120.90	0.00	1876.47

### Evaluation of differentially modified H3K27me3 regions

#### qPCR validation of selected regions

**Figure 3:**
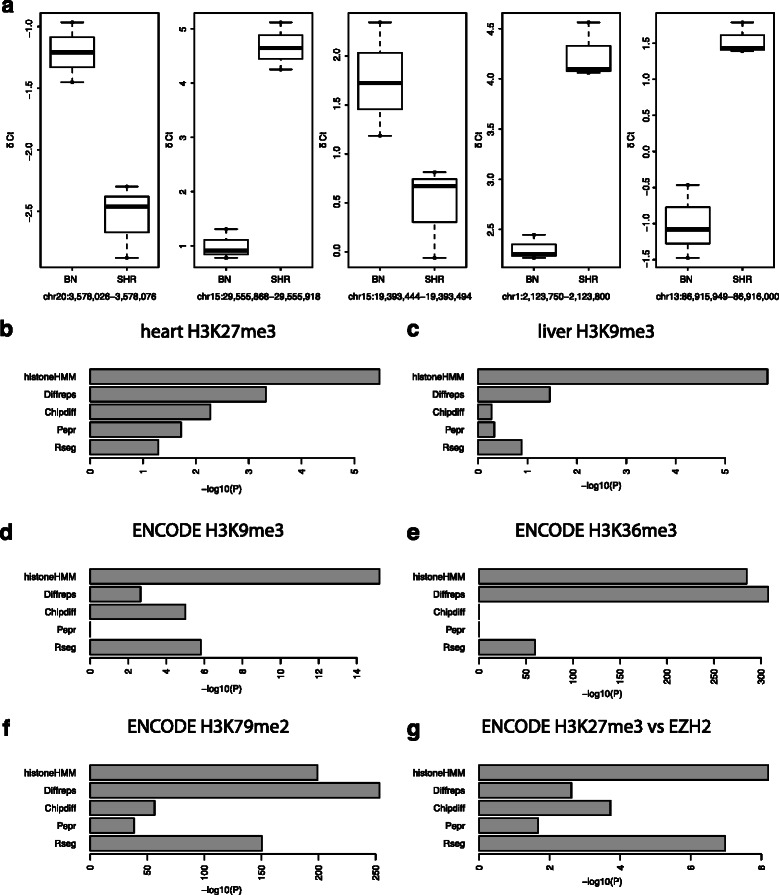
**Evaluation of differentially modified regions.** **a)** qPCR validation of differential H3K27me3 regions in rat heart tissue. The boxplots show the level of histone modifications for the five true positive regions that were not overlapping genomic deletions. Modification levels are measured as *δ*CT values for the two rat strains BN and SHR. All differences are significant (t-test *P*<0.05). **b-f)** Barplots of −*l* *o* *g*_10_(*P*) of Fisher’s exact test for overlap of differentially modified genes with differentially expressed genes for each of the methods. **b)** shows results for H3K27me3 in the rat strain comparison. **c)** shows results for H3K9me3 in the comparison of female and male mice. **d-f)** show results of the comparison of ENCODE cell lines H1-hESC and K562. Differential H3K9me3, H3K36me3 and H3K79me2 regions are compared to differential gene expression. **g)** shows results of the comparison of ENCODE cell lines H1-hESC and K562. Differential H3K27me3 regions are compared to genes with differential EZH2 levels.

qPCR analysis was carried out on 11 regions that were called differentially modified by histoneHMM between SHR and BN, and had a read count fold-change of larger than two (Table 3). For 4 of these regions we detected no amplification signal in the SHR strain. Further analysis showed that these regions overlapped genomic deletions in SHR and are therefore not genuine differentially modified regions. Nonetheless, since these deletions produce differential ChIP-seq signals, we consider these histoneHMM calls as true positives. Of the remaining 7 regions all but 2 were confirmed by qPCR (Figure 3a). For comparison, Chipdiff and Rseg were only able to detect 5 and 6 of the validated differential regions, respectively, suggesting a higher false negative rate relative to histoneHMM, at least for the limited number of regions surveyed here. Diffreps performed similar to histoneHMM. It detected all qPCR validated differential regions, but also predicted the same two regions that could not be validated using qPCR.

**Table 3:** **Detection of qPCR validated H3K27me3 regions in the rat**

**Region**	**Chrom**	**Start**	**End**	**qPCR validation**	**deletion**	**histoneHMM**	**Diffreps**	**Chipdiff**	**Rseg**
1	chr5	108,178,675	108,178,725	diff	Y	Y	Y	FN	Y
2	chr20	3,578,026	3,578,076	diff	N	Y	Y	Y	Y
3	chr20	4,476,835	4,476,885	non-diff	N	FP	FP	FP	Y
4	chr20	4,677,234	4,677,284	diff	Y	Y	Y	Y	Y
5	chr15	29,555,868	29,555,918	diff	N	Y	Y	FN	Y
6	chr11	76,487,730	76,487,780	non-diff	N	FP	FP	FP	FP
7	chr15	19,393,444	19,393,494	diff	N	Y	Y	Y	FN
8	chr1	2,026,376	2,026,436	diff	Y	Y	Y	FN	FN
9	chr1	2,123,750	2,123,800	diff	N	Y	Y	FN	FN
10	chr13	86,915,949	86,916,000	diff	N	Y	Y	Y	Y
11	chr15	30,003,020	30,003,070	diff	Y	Y	Y	Y	Y

“diff” stands for validated differential enrichment, and “non-diff” for validated non differential enrichment. “deletion” indicated whether the region overlaps with a genomic deletion in the SHR strain. FP = False Positives; FN = False Negatives; Y = correctly detected.

#### RNA-seq analysis of differentially modified H3K27me3 regions

Because the number of regions used for qPCR validation was small and biased towards our method (only regions called by histoneHMM were selected), we performed additional functional validation of differential calls using RNA-seq data from age-matched animals (Table 1).

We employed DESeq [9] to identify genes that are differentially expressed between SHR and BN, and assessed the overlap between these genes and the set of differentially modified regions detected by each of the methods. Our results show that histoneHMM yielded the most significant overlap (*P*=3.36×10^−6^, Fisher’s exact test, Figure 3b).

The genes that were concordantly differentially expressed and differentially modified are plausible causal candidates for hypertension in SHR. Gene ontology analysis revealed enrichment for the GO term “antigen processing and presentation” (GO:0019882, *P*= 4.79·10^−7^). These were mainly genes from the MHC class I complex which is a key part of the innate immune response. Interestingly, all of the differential MHC genes are located in blood pressure quantitative trait loci (QTL) that were previously identified using either crosses derived from these two strains or from closely related strains [22]. Integration of our ChIP-seq results with these QTL mapping data can thus help prioritize targets within the QTL intervals for experimental follow-up.

#### Comparison of differential H3K27me3 regions and differential polycomb binding

H3K27me3 is a hallmark of repression by the polycomb complex [1,11]. The genome wide binding patterns of EZH2, a major component of the polycomb complex, has been characterized in the human embryonic stem cell line H1-hESC (H1) as well as in the K562 cell line by the ENCODE project. EZH2 is characterized by a similarly broad pattern as H3K27me3. Since H3K27me3 is deposited by the polycomb complex it is expected that differential H3K27me3 occupancy between cell lines is related to differential EZH2 binding. In order to be able to compare the two differential signals without having to rely on a segmentation algorithm for the EZH2 data, we quantified EZH2 occupancy on gene bodies. Subsequently we identified genes with differential EZH2 read counts using DESeq (*F* *D* *R*<0.01). In analogy to the comparison with differential gene expression, we assessed the significance of the overlap of differential EZH2 genes with differential H3K27me3 region calls. Figure 3g shows that histoneHMM yielded the most significant overlap, indicating that differential H3K27me3 calls are biologically relevant.

### Evaluation of differentially modified H3K9me3 regions

#### Validation using known X-chromosome inactivated genes

Inactivation of one copy of the X chromosome in female mammals is a well characterized mechanism of dosage compensation [23]. Early cytogenetic observations showed that one copy is in a heterochromatic state [24] while the other copy remains accessible. H3K9me3 is one of the hallmarks of heterochromatin [11], therefore inactivated regions are expected to be called differentially modified between female and male mice. We obtained a high confidence list of 250 X inactivated protein coding genes [25] and determined the percentage of basepairs of these genes that was called differentially modified by each of the methods studied here. Table 4 shows that histoneHMM correctly identifies 62% of inactivated basepairs as differentially modified corresponding to 99% of inactivated genes, which is substantially more than what is reported by Diffreps, Chipdiff and Pepr.

**Table 4:** **Percentage of base pairs (% bp) from X inactivated genes that are called differentially enriched for H3K9me3 between male and female mice and percentage of X inactivated genes that overlap with at least 1bp of differentially enriched regions (% genes)**

	**histoneHMM**	**Diffreps**	**Chipdiff**	**Pepr**	**Rseg**
% bp	67%	06%	10%	0.0	100%
% genes	99%	84%	78%	0.0	100%

Interestingly, Rseg appears to call 100% of the inactivated basepairs in these data. However, the very large number of basepairs predicted exclusively by Rseg (Figure 2) and the poor overlap with differential gene expression (Figure 3) suggests that this is likely a consequence of Rseg overpredicting large parts of the genome as differentially modified.

#### RNA-seq analysis of differentially modified H3K9me3 regions

We further explored the relationship between differential enrichment for H3K9me3 and genome-wide gene expression differences between male and female mice. Similar to the RNA-seq analysis discussed above, we obtained differentially expressed genes provided in [16], and then assessed the overlap between these genes and the set of differentially modified H3K9me3 regions detected by each of the methods. Again, histoneHMM yielded the most significant overlap (*P*=1.39×10^−6^, Fisher’s exact test, Figure 3c).

The expression differences between sexes in liver is of particular interest for toxicology because many cytochrome P450 (Cyp) genes involved in drug metabolism are affected [26]. It has been shown that liver gene expression of *Cyp2b9* and *Cyp2a4* in females can permanently be changed from a female to a male program by a single application of testosterone early in life, however for *Cyp2d9* this is not the case [27]. Using histoneHMM, we found that *Cyp2d9* is fully contained in a H3K9me3 domain specifically in females but partly unmodified in males, while the other two genes are partly unmodified in both sexes and do not show sex specific modifications. Thus the differences of hormone activation between *Cyp2d9* on the one side and *Cyp2b9* and *Cyp2a4* on the other, could be due to the female specific heterochromatic state of *Cyp2d9*.

### Evaluation of differential H3K36me3, H3K79me2 and H3K9me3 calls in ENCODE cell lines

We evaluated the performance of differential peak calling tools on additional histone modifications from the ENCODE cell lines H1-hESC (H1) and K562. We investigated H3K36me and H3K79me2 that are related to active transcription and occur preferentially in gene bodies. We also included the H3K9me3 data set in order to corroborate the results obtained on the mouse data, which had a relatively low read coverage (see Table 1). We were mainly interested to assess how versatile the compared methods are and to identify potential biases of any method towards certain histone modifications.

For the evaluation we again compared the differentially called regions to differential gene expression, that was obtained from ENCODE RNA-seq data. Figure 3d shows that histoneHMM outperforms the other tools for H3K9me3 also in the ENCODE cell lines and thereby confirms the results based on the mouse data set. Figure 3e-f shows the performance for H3K36me3 and H3K79me2. It is worth noting that the relation between differential gene expression and differential histone modifications is much more pronounced for H3K36me and H3K79me2 than for H3K27me3 or H3K9me3 since the former are directly related to the transcriptional process.

The results show that histoneHMM is an efficient algorithm for detecting functionally relevant differentially modified regions. This is likely due to an overall lower false positive and false negative rate. Indeed, extensive simulation studies support this conclusion (Additional file 1).

### Runtime evaluation

**Figure 4:**
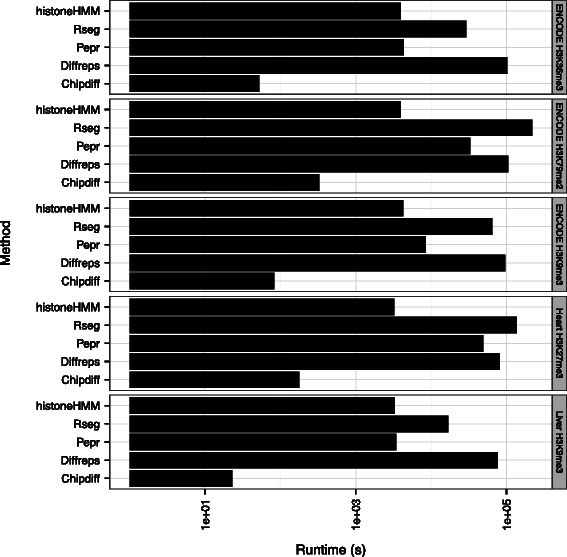
**Runtime evaluation.** Runtimes are given in seconds user time on a 1500 MHz AMD processor. Note that the scale is logarithmic.

We evaluated the runtime of each algorithm on each of the data sets presented above. We measured the user time on a 1150 MHz Quad-Core AMD Opteron Processor 2356. Figure 4 shows that Chipdiff is the fastest algorithm on all data sets, followed by histoneHMM. Note that the figure has a log scale, so other algorithms are orders of magnitude slower.

### Application of histoneHMM to single ChIP-seq samples

Although histoneHMM was primarily designed for the detection of differentially modified regions between two ChIP-seq samples, it can also be easily applied to the analysis of a single ChIP-seq sample. In this case histoneHMM classifies the genome into regions that are modified or unmodified. Analysis of single ChIP-seq samples is common practice and many algorithms have been developed for this purpose [28-32]. However, analyzing ChIP-seq data with broader genomic footprints is still challenging. We compared the performance of histoneHMM to several popular peak callers that were specifically developed for that task: Macs2 with the broad option [28], Zinba [33], Sicer [34], Broadpeak [35] and Rseg [21].

**Figure 5:**
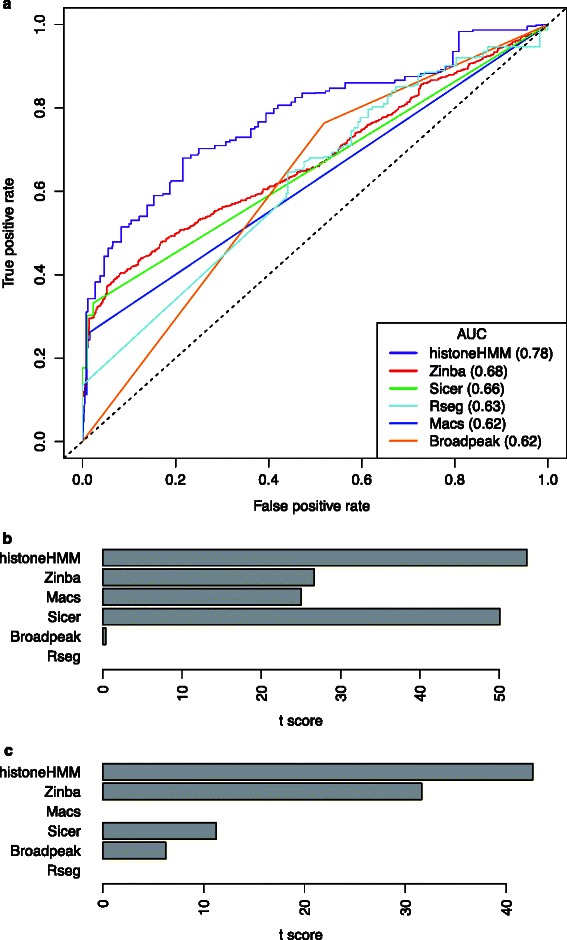
**Single ChIP-seq sample analysis.** **a)** Receiver operator characteristics curves show the sensitivity and specificity of different methods for H3K27me3 peak calls evaluated using qPCR validated regions. **b-c)** The association of region calls with gene expression is quantified using the *t*-statistic of gene expression values when comparing expression levels of genes with at least 50% overlap with modified regions to genes with less overlap. The barplots show the results for **b)** H3K27me3 and **c)** H3K9me3. Macs and Rseg have missing values because macs did not yield any gene with at least 50% overlap for H3K9me3, while Rseg did not yield any gene with less than 50% overlap for both modifications.

For this comparative analysis, we used a publicly available data set of qPCR validated H3K27me3 regions, which was previously used by Micsinai et al. [36] for a similar purpose. It consists of a ChIP-seq and a input control data set for normalization (GEO accessions GSM721294, GSM721306) and a set of 197 regions with positive or negative qPCR validation status. This data set is ideal as it allows for the calculation of the sensitivity and specificity of each method. Following Micsinai et al. [36] we considered each basepair in the qPCR validated regions as a data point and labeled it zero if it was not enriched and one if it was enriched. The corresponding ChIP-seq data was then analyzed using the standard settings of each peak caller, and each base pair in the genome was assigned a score (e.g. latent state probability or *P*-value) by the algorithm. Since most peak callers do not return basepair resolution results, predictions for each basepair were obtained by taking the peak caller’s result in the interval overlapping the basepair position. It is worth noting that our evaluation differs from the one of Micsinai et al. because their score depends on the full set of all predictions that are to be compared. Since we have used a different set of predictions including those of our own tool the results are not directly comparable. In addition, the authors computed the AUCROC by setting a fixed threshold for each method, and therefore did not use the full potential of ROC analysis which measures the performance across the full range of possible threshold values. With this in mind, our sensitivity-specificity analysis revealed that histoneHMM outperforms the other algorithms in the detection of modified versus unmodified regions (Figure 5a).

In addition we used gene expression data to evaluate calls of modified and unmodified regions functionally. In particular this allowed us to evaluate the performance of the univariate HMM for both the rat H3K27me3 (BN) and mouse H3K9me3 (female) data set, as for the latter no qPCR data was available. Regions that were called modified with H3K27me3 or H3K9me3 had repressed gene expression compared to regions that were called unmodified. The differences in gene expression were more pronounced for histoneHMM compared to the other methods (Figure 5b-c). As argued above, this results is likely the outcome of lower false positive and false negative rates.

## Conclusions

The comparison of two ChIP-seq samples is an important tool to identify genes and regulatory mechanisms involved in diverse biological processes. While several algorithms exist for peak-like modifications (e.g. [9,10]), they are known to perform poorly for broad marks such as the heterochromatin-associated H3K27me3 and H3K9me3. Here we introduced histoneHMM, a powerful Hidden Markov Model for the comparison of ChIP-seq profiles between two samples. Using real and synthetic data, we demonstrated that histoneHMM outperforms competing methods in the detection of differentially modified regions.

histoneHMM is not limited to this bivariate setting but can, in principle, be extended to an arbitrary number of dimensions. This feature makes it possible to compare a large number of samples for the same histone mark, or alternatively, many different histone marks in a single sample (e.g. in a single cell line). This latter application provides a formal method to detect and characterize combinatorial histone states in a genome-wide manner. Extension to histoneHMM to perform such a combinatorial analysis promises to provide a powerful alternative to chromHMM or Segway, two algorithms that have been employed in the ENCODE project.

histoneHMM runs in the popular R computing environment and integrates with the extensive bioinformatic tool sets available through Bioconductor. This makes histoneHMM an attractive choice for the differential analysis of ChIP-seq data.

## Methods

### ChIP-seq data

Histone modification H3K27me3 was analyzed using ChIP-seq in the left ventricle of the heart from 3 biological replicates of each rat strain BN and SHR (Array-Express [37] accession number E-MTAB-1102). All ChIP-seq reads were aligned to the rat genome rn4 using Eland v2 (Illumina CASAVA 1.7). In order to avoid differential read counts that may arise due to increased number of mismatches when aligning to positions with known sequence variation in the non-reference strain SHR, we aligned SHR samples against the BN reference genome with known SNP positions [38] substituted by the SHR alleles.

The ChIP-seq data from mouse myoblast cells that constitutes the H3K27me3 benchmark data set [36] was downloaded from gene expression omnibus (GEO accessions GSM721294, GSM721306).

H3K9me3 data from livers of male and female mice from [16] was downloaded from the European nucleotide archive (ENA accession SRP018808).

ChIP-seq data from ENCODE was downloaded from the UCSC ENCODE data center (GEO sample accession numbers: GSM1003585, GSM1003585, GSM733748, GSM733748, GSM733725, GSM733725, GSM1003547, GSM1003547, GSM733658, GSM733658, GSM733714, GSM733714, GSM733653, GSM733653, GSM733776, GSM733776, GSM1003524, GSM1003576). We used the aligned reads (genome assembly hg19) resulting from the uniform processing pipeline of ENCODE and removed duplicated reads using samtools. Since the coverage of H3K27me3 was very low for H1-hESC (Table 1, H1 H3K27me3 replicates 1-2) we obtained additional data for H1-hESC H3K27ne3 from the roadmap epigenomics [39] project (SRA accession numbers: SRR019561, SRR029343, SRR029345, SRR029347, SRR029349).

All histone marks analyzed here are characterized by broad genomic footprints. Therefore, coverage is relatively low and we used binning to aggregate data from larger regions. As input for our HMM we counted start positions of all uniquely mapping reads, after removal of duplicated reads. In order to avoid artifacts from regions with extreme read counts [40] and to avoid numerical problems with very small emission probabilities we truncated read counts at the upper 0.1 percentile. All read counts greater than the upper 0.1 percentile were set to the value of the upper 0.1 percentile.

### Gene expression data

Gene expression levels were measured using RNA-seq in the left ventricle of the heart from 5 animals per strain, which were matched to the animals used for ChIP-seq for age and sex (Array-Express accession number E-MTAB-1102). Reads were mapped to the BN reference genome rn4 using TopHat v 1.2.0. [41]. Gene expression levels were estimated by counting reads corresponding to exons of protein coding genes from Ensembl release 59. For the comparison of gene expression within a sample, expression levels were normalized to the length of the gene. Differential expression between strains was determined from the unnormalized read counts using the DESeq method [9] with *F* *D* *R*<0.01.

Liver gene expression data for the comparison of female and male mice was obtained from gene expression omnibus (GEO accession GSE48109). This data also comprises differential gene expression results obtained by the authors using edgeR [42].

ENCODE RNA-seq data for H1-hESC and K562 cell lines (GEO accession: GSM758566, GSM765405) was obtained from the UCSC ENCODE data center. Here we also used the aligned reads (hg19) as proccessed by the ENCODE pipeline. We obtained read counts as measure of gene expression using gene annotation from ENSEMBL release 63. Differential gene expression was determined using the DESeq method [9] with *F* *D* *R*<0.01.

### Model specifications

#### Univariate Hidden Markov Model

For a single ChIP-seq sample, we partition the genome into *m* equally sized bins (1000 bp by default). Let *x*_*i*_ be the read counts for the *i*th bin. We model the density of *x*_*i*_ as a two-component finite mixture. The mixture is characterized by a heavy tail, indicating a modified component, as well as by a concentration of probability mass at low counts, especially at zero, corresponding to non-enriched regions. We write the density as

(1) $$  P(x|\mathbf{\theta}) = \alpha f(x, \theta_{0}) + (1 - \alpha) f(x, \theta_{1}),  $$

where *α* is the mixing weight and *θ*_0_ and *θ*_1_ are the component density parameters. Following others [33], we assume that each mixing component is given by a zero-inflated negative binomial distribution (zinb), hence, for the *j*th component the density is

(2) $$  f(x, \theta_{j}=(r, p, \beta)) = \beta I_{x = 0} + (1 - \beta) \frac{\Gamma(r + x)}{\Gamma(r)x!} p^{r}(1-p)^{x},  $$

where *Γ* denotes the gamma function, *I*_*x*=0_ is an indicator function and *β* is the inflation parameter for zero counts. *p* and *r* are the probability and the dispersion parameter of the negative binomial distribution, respectively. Without loss of generality we assume that state 0 represents the low occupancy values (*μ*_0_<*μ*_1_). Parameter estimates are obtained via the EM algorithm [43]. We obtained starting values for the EM by partitioning the data into two groups at the median. The group with counts less than the median was assigned probability 0.9 to be from the first mixture component and 0.1 to be from the second and vice versa for the second group. Then the parameters of the mixture components were updated just as in the maximization step of the EM algorithm. For improved runtime efficiency we used only data from one chromosome (chr18) for the parameter estimation.

To analyze single ChIP-seq samples we use the unmodified and the modified component of this mixture as fixed emission densities in a univariate HMM with two states, unmodified and modified respectively. We use the Baum-Welch algorithm [44] to determine the transition probabilities between states, and calculate the probability of enrichment for each bin in the genome using the forward-backward algorithm [45]. Chromosomes were processed one by one using the same fixed emission probabilities. We called bin *j* modified when the latent state probability of being enriched in this bin is greater than a certain threshold *λ*. If not otherwise stated we used *λ*=0.5, which corresponds to the latent state with maximal probability in the two state model. Simulation studies showed that this parameter setting yields good sensitivity and specificity (Additional file 1).

Alternatively, the parameter estimates for this two-component mixture can be trained using gene-expression data (Additional file 1). Since H3K27me3 and H3K9me3 modifications are associated with gene silencing, the heavy tail with high occupancy values can be associated with lowly expressed genes and the low occupancy counts with highly expressed genes. Using gene expression increased the performance of the algorithm (Additional file 1), both for the single sample analysis and for the sample comparison.

#### Bivariate hidden Markov model

histoneHMM is primarily designed to compare two ChIP-seq samples, say *A* and *B*. For each individual ChIP-seq sample, we partition the genome into *m* equally sized bins (1000 bp by default). Let *x*_*i*_ and *y*_*i*_ be the read counts for the *i*th bin for sample *A* and *B*, respectively. Further we define the indicator variable *a*=0 if sample *A* is unmodified and *a*=1 if it is modified. Similarly the indicator variable *b* is defined for sample *B*. We denote the parameters of the univariate mixture of sample *A* as *θ*_*A*_ and that of sample *B* as *θ*_*B*_. The probability of the random pair (*x*_*i*_,*y*_*i*_) is given by a bivariate count distribution with four mixing components, corresponding to the situations where both samples are unmodified (*a*=0,*b*=0), both samples are modified (*a*=1,*b*=1), only sample *A* is modified (*a*=1,*b*=0) or only sample *B* is modified (*a*=0,*b*=1). We write this four component mixture as

(3) $$  P((x,y)|\mathbf{\theta}) = \sum_{a=0}^{1} \sum_{b=0}^{1}{\gamma_{a,b} f((x,y),\theta_{a,b})},  $$

where *γ*_*a*,*b*_ are the mixing weights and *θ*_*a*,*b*_ are the component density parameters for each component *j*, corresponding to a pair *a*,*b*.

Calculating the bivariate components *f*((*x*,*y*),*θ*_*a*,*b*_) is challenging as bivariate (or multivariate) count distributions are difficult to work with and often do not exist in closed form. Copula theory offers an elegant way to obtain multivariate distributions once the marginals are known [46]. A copula *C*=*C*(*u*_1_,*u*_2_,…,*u*_*p*_)=*P*(*U*_1_≤*u*_1_,*U*_2_≤*u*_2_,…,*U*_*p*_≤*u*_*p*_) is a multivariate cumulative density function (CDF) defined over the *p*-dimensional unit cube *C*:[0,1]^*p*^→[0,1], where each *U*_*i*_∼Unif(0,1). For two random variables *Z*_*x*_,*Z*_*y*_ with joint CDF *G* and marginal CDFs *G*_*x*_,*G*_*y*_ the probability integral transformation can be used to obtain a copula $C(u_{x},u_{y}) = G(G_{x}^{-1}(u_{x}), G_{y}^{-1}(u_{y}))$. Here we used a Gaussian copula, such that *G* is the CDF of the multivariate Normal distribution and *G*_*x*_,*G*_*y*_ are the corresponding univariate Normal marginal CDFs. To obtain a CDF for the original random variables *X* and *Y* with marginal CDFs ${F^{a}_{x}}$ and ${F^{b}_{y}}$ we use again the probability integral transformation to obtain the uniform variables $u_{x}={F^{a}_{x}}(x)$ and $u_{y}={F^{b}_{y}}(y)$. For a more detailed introduction to copula theory we refer the reader to [47]. Now putting it all together, we used a Gaussian copula to define the bivariate cumulative distribution function of each component *F*((*x*,*y*),*θ*_*a*,*b*_) as

$$\begin{aligned} C^{a,b}\left({F^{a}_{x}}(x), {F^{a}_{y}}(y)\right) &= P\left(X \leq x, Y \leq y\right)\\ & = \Phi_{\Sigma_{a,b}} \left(\Phi^{-1}\left({F^{a}_{x}}(x)\right), \Phi^{-1}\left({F^{b}_{y}}(y)\right)\right)\!, \end{aligned} $$

where

$$ \begin{aligned} \Phi_{\Sigma_{a,b}}\left(z_{x}, z_{y}\right) & = \int_{-\infty}^{z_{x}} \int_{-\infty}^{z_{y}} \phi_{\Sigma_{a,b}} \left(z_{x}, z_{y}\right) {dz}_{x}{dz}_{y}, \\ \phi_{\Sigma_{a,b}}\left(z_{x}, z_{y}\right) & = \frac{1}{2 \pi \sigma_{x} \sigma_{y} \sqrt{1 - \rho^{2}}}\\ &\quad\times\exp\!\left(\frac{-1}{2 (1 - \rho^{2})}\left[\frac{{z_{x}^{2}}}{{\sigma_{x}^{2}}} + \frac{{z_{y}^{2}}}{{\sigma_{y}^{2}}} - \frac{2 \rho z_{x} z_{y}}{\sigma_{x} \sigma_{y}} \right]\right) \end{aligned} $$

is the bivariate Gaussian CDF with zero mean and covariance matrix corresponding to *ρ*,*σ*_*x*_,*σ*_*y*_. *Φ* ^−1^ is the inverse of the univariate standard normal CDF and ${F^{a}_{x}} = P(X \leq x) = {\sum _{0}^{x}} f(x, \theta _{A,a})$ is the CDF for the marginal distribution of component *a* of sample *A*, and ${F^{b}_{y}} = P(Y \leq y) = {\sum _{0}^{y}} f(y, \theta _{B,b})$ for the component *b* of *B*, respectively (Eq. 1, Eq. 2).

The covariance matrix *Σ*_*a*,*b*_ between the transformed variables $\Phi ^{-1}({F^{a}_{x}}(x))$ and $\Phi ^{-1}({F^{b}_{y}}(y))$ is computed as follows: first we called each region modified or unmodified in samples *A* and *B* separately using the univariate HMM approach outlined above. We used regions that had high confidence calls (with latent state probability >0.9) in both samples and created four subsets of regions for all possible combinations of univariate states (*a*,*b*). Then for every given subset the read data (*x*,*y*) was transformed to $(z_{x}, z_{y}) = \left (\Phi ^{-1}\left ({F^{a}_{x}}(x)\right), \Phi ^{-1}\left ({F^{b}_{y}}(y)\right)\right)$ using the marginal distributions *f*(*x*,*θ*_*A*,*a*_) and *f*(*y*,*θ*_*B*,*b*_). Finally *Σ*_*a*,*b*_ was estimated by the sample covariance of the transformed data in each subset.

Since we are working with discrete count data we are interested in the probabilities

(4) $$   \begin{aligned} P(X=x, Y=y) & \!\!= F((x, y))\! -\! F((x-1, y)) - F((x, y-1)) \\ & \quad + F((x-1, y-1))\\ & \,=\,\! \int_{\Phi^{\,-\,1}(F_{x}(x\,-\,1))}^{\Phi^{\,-\,1}(F_{x}(x))} \!\int_{\Phi^{\,-\,1}(F_{y}(y\,-\,1))}^{\Phi^{\,-\,1}(F_{y}(y))}\phi_{\Sigma} (z_{x}, z_{y}) {dz}_{x}{dz}_{y}. \end{aligned}  $$

We evaluate this integral using numerical integration techniques [48].

Having defined this bivariate count distribution we proceed to construct a HMM for the identification of differentially modified regions between samples *A* and *B*. This HMM has four states, corresponding to the situations where both samples are unmodified, both samples are modified, only sample *A* is modified or only sample *B* is modified. The four fixed emission densities are given by the four components of the bivariate mixture (Eq. 3), respectively and are evaluated according to Eq. 4. Transitions from all states to all other states as well as self transitions are allowed. We use the Baum-Welch algorithm to estimate the transition probabilities and we classify each bin into one of the four states using the maximal latent state probability obtained by the forward-backward algorithm.

### Region calling with other methods

In this section we describe how the other tools in the comparison were run. When possible we always set the bin size to 1000 bp. We mostly used the default parameters and thresholds as recommended by the authors since these results are likely those that an end user would also obtain. In order to rule out that the results of our comparisons are biased by the choice of threshold described here, we also performed a systematic evaluation of thresholds to optimize the performance of each individual method (Additional file 1).

#### Differential region calling

##### Chipdiff

We ran Chipdiff with a maximum of 500 iterations and 10000 training sequences. As recommended by the authors we used a minimal fold change of 2, but we also tried other thresholds in our simulation study (see Additional file 1). We used the threshold of latent state probability *P*>0.95 to call differential regions.

##### Rseg

We used the ‘rseg-diff’ software with ‘-mode 3’. Since rseg is also based on a HMM we obtained latent state probabilities for differential regions for all bins in the genome. Finally we used the same threshold *P*>0.5 that we used for histoneHMM to call differential regions.

##### Diffreps

We used Diffreps without replicates using the chi squared test (‘-meth cs’). As recommended by the authors we used a threshold of *P*<0.0001 on the *P*-value to call differential regions. We did not use DNA input or IgG controls.

##### Pepr

Pepr is the only tool in the comparison that works only when replicates are provided, so we used all available replicates before merging them. The authors recommend a threshold of *P*<10^−5^ on the *P*-value.

#### Region calling in single samples

In this comparison we always used input control data when possible.

##### Macs

We used macs version 2 with the broad option. The recommended threshold for peak calling was *F* *D* *R*<0.01.

##### Zinba

We used the mappability files for human, mouse and rat that were provided on the Zinba website. We used the generalized linear model with just the input count as predictor. For the ROC analysis we used the latent state probability of modification. For the comparison to gene expression we used the threshold *P*>0.5 on the latent state probability to call regions.

##### Sicer

Sicer was run with a window size of 200 bp and a gap size of 600 bp as recommended for H3K27me3 by the authors. Significant regions were identified using *F* *D* *R*<0.01.

##### Broadpeak

Broadpeak does not output scores and also does not require the specification of a threshold, therefore we just ran Broadpeak with default options and used all predictions that were returned.

##### Rseg

We used the ‘rseg-diff’ software with ‘-mode 2’ to provide the input control data. We obtained latent state probabilities for modified regions for all bins in the genome. Finally we used the same latent state probability threshold *P*>0.5 that we used for histoneHMM to call regions.

### Software

The software was implemented in the R package *histoneHMM* and is freely available from http://histonehmm.molgen.mpg.de.

## Additional file

Additional file 1
**Supplemental information.**

## References

[1] Kouzarides T (2007). Chromatin modifications and their function. Cell.

[2] Beck DB, Oda H, Shen SS, Reinberg D (2012). PR-Set7 and H4K20me1: at the crossroads of genome integrity, cell cycle, chromosome condensation, and transcription. Genes Dev.

[3] Huda A, Mariño-Ramírez L, Jordan IK (2010). Epigenetic histone modifications of human transposable elements: genome defense versus exaptation. Mob DNA.

[4] Pengelly AR, Ömer C, Jäckle H, Herzig A, Müller J (2013). A histone mutant reproduces the phenotype caused by loss of histone-modifying factor Polycomb. Science.

[5] Chi P, Allis CD, Wang GG (2010). Covalent histone modifications–miswritten, misinterpreted and mis-erased in human cancers. Nat Rev Cancer.

[6] Peleg S, Sananbenesi F, Zovoilis A, Burkhardt S, Bahari-Javan S, Agis-Balboa RC (2010). Altered histone acetylation is associated with age-dependent memory impairment in mice. Science.

[7] Reuter S, Gupta SC, Park B, Goel A, Aggarwal BB (2011). Epigenetic changes induced by curcumin and other natural compounds. Genes Nutr.

[8] Park PJ (2009). ChIP-seq: advantages and challenges of a maturing technology. Nat Rev Genet.

[9] Anders S, Huber W (2010). Differential expression analysis for sequence count data. Genome Biol.

[10] Shao Z, Zhang Y, Yuan G-C, Orkin S, Waxman D (2012). MAnorm: a robust model for quantitative comparison of ChIP-Seq data sets. Genome Biol.

[11] Beisel C, Paro R (2011). Silencing chromatin: comparing modes and mechanisms. Nat Rev Genet.

[12] Barski A, Cuddapah S, Cui K, Roh T-Y, Schones DE, Wang Z (2007). High-resolution profiling of histone methylations in the human genome. Cell.

[13] Mikkelsen T, Ku M, Jaffe D, Issac B, Lieberman E, Giannoukos G (2007). Genome-wide maps of chromatin state in pluripotent and lineage-committed cells. Nature.

[14] Okamoto K (1972). Spontaneous Hypertension: Its Pathogenesis and Complications.

[15] Rintisch C, Heinig M, Bauerfeind A, Schafer S, Mieth C, Patone G (2014). Natural variation of histone modification and its impact on gene expression in the rat genome. Genome Res.

[16] Sugathan A, Waxman DJ (2013). Genome-wide analysis of chromatin states reveals distinct mechanisms of sex-dependent gene regulation in male and female mouse liver. Mol Cell Biol.

[17] E.N.C.O.D.E Project Consortium (2012). An integrated encyclopedia of DNA elements in the human genome. Nature.

[18] Shen L, Shao N-Y, Liu X, Maze I, Feng J, Nestler EJ (2013). diffReps: detecting differential chromatin modification sites from ChIP-seq data with biological replicates. PLoS One.

[19] Xu H, Wei C-L, Lin F, Sung W-K (2008). An HMM approach to genome-wide identification of differential histone modification sites from ChIP-seq data. Bioinformatics.

[20] Zhang Y, Lin Y-H, Johnson TD, Rozek LS, Sartor MA (2014). PePr: a peak-calling prioritization pipeline to identify consistent or differential peaks from replicated ChIP-Seq data. Bioinformatics.

[21] Song Q, Smith AD (2011). Identifying dispersed epigenomic domains from ChIP-Seq data. Bioinformatics.

[22] Dwinell MR, Worthey EA, Shimoyama M, Bakir-Gungor B, DePons J, Laulederkind S (2009). The rat genome database 2009: variation, ontologies and pathways. Nucleic Acids Res.

[23] Augui S, Nora EP, Heard E (2011). Regulation of X-chromosome inactivation by the X-inactivation centre. Nat Rev Genet.

[24] Lyons MF (1961). Gene action in the X-chromosome of the mouse (Mus musculus L.). Nature.

[25] Yang F, Babak T, Shendure J, Disteche CM (2010). Global survey of escape from X inactivation by RNA-sequencing in mouse. Genome Res.

[26] Rinn JL, Rozowsky JS, Laurenzi IJ, Petersen PH, Zou K, Zhong W (2004). Major molecular differences between mammalian sexes are involved in drug metabolism and renal function. Dev Cell.

[27] Ramirez MC, Luque GM, Ornstein AM, Becu-Villalobos D (2010). Differential neonatal testosterone imprinting of GH-dependent liver proteins and genes in female mice. J Endocrinol.

[28] Zhang Y, Liu T, Meyer CA, Eeckhoute J, Johnson DS, Bernstein BE (2008). Model-based analysis of ChIP-Seq (MACS). Genome Biol.

[29] Kharchenko PV, Tolstorukov MY, Park PJ (2008). Design and analysis of ChIP-seq experiments for DNA-binding proteins. Nat Biotechnol.

[30] Spyrou C, Stark R, Lynch AG, Tavaré S (2009). BayesPeak: Bayesian analysis of ChIP-seq data. BMC Bioinformatics.

[31] Qin ZS, Yu J, Shen J, Maher CA, Hu M, Kalyana-Sundaram S (2010). HPeak: an HMM-based algorithm for defining read-enriched regions in ChIP-Seq data. BMC Bioinformatics.

[32] Cairns J, Spyrou C, Stark R, Smith ML, Lynch AG, Tavaré S (2011). BayesPeak–an R package for analysing ChIP-seq data. Bioinformatics.

[33] Rashid NU, Giresi PG, Ibrahim JG, Sun W, Lieb JD (2011). ZINBA integrates local covariates with DNA-seq data to identify broad and narrow regions of enrichment, even within amplified genomic regions. Genome Biol.

[34] Zang C, Schones DE, Zeng C, Cui K, Zhao K, Peng W (2009). A clustering approach for identification of enriched domains from histone modification ChIP-Seq data. Bioinformatics.

[35] Wang J, Lunyak VV, Jordan IK (2013). BroadPeak: a novel algorithm for identifying broad peaks in diffuse ChIP-seq datasets. Bioinformatics.

[36] Micsinai M, Parisi F, Strino F, Asp P, Dynlacht BD, Kluger Y (2012). Picking ChIP-seq peak detectors for analyzing chromatin modification experiments. Nucleic Acids Res.

[37] Parkinson H, Kapushesky M, Shojatalab M, Abeygunawardena N, Coulson R, Farne A (2007). ArrayExpress–a public database of microarray experiments and gene expression profiles. Nucleic Acids Res.

[38] Atanur SS, Birol I, Guryev V, Hirst M, Hummel O, Morrissey C, et al.The genome sequence of the spontaneously hypertensive rat: Analysis and functional significance. Genome Res. 2010. doi:10.1101/gr.103499.109. 10.1101/gr.103499.109 PMC2877576 20430781

[39] Bernstein BE, Stamatoyannopoulos JA, Costello JF, Ren B, Milosavljevic A, Meissner A (2010). The NIH roadmap epigenomics mapping consortium. Nat Biotechnol.

[40] Carroll TS, Liang Z, Salama R, Stark R, de Santiago I (2014). Impact of artifact removal on ChIP quality metrics in ChIP-seq and ChIP-exo data. Front Genet.

[41] Trapnell C, Pachter L, Salzberg SL (2009). TopHat: discovering splice junctions with RNA-seq. Bioinformatics.

[42] Robinson MD, McCarthy DJ, Smyth GK (2010). edgeR: a Bioconductor package for differential expression analysis of digital gene expression data. Bioinformatics.

[43] Dempster AP, Laird NM, Rubin DB (1977). Maximum likelihood from incomplete data via the EM algorithm. J R Stat Soc Ser B (Methodological).

[44] Baum L, Petrie T, Soules G, Weiss N (1970). A maximization technique occurring in the statistical analysis of probabilistic functions of markov chains. Ann Math Stat.

[45] Rabiner L (1989). A tutorial on hidden Markov models and selected applications in speech recognition. Proc IEEE.

[46] Sklar A (1959). Fonctions de répartition à n dimensions et leurs marges. Publ Inst Statist Univ Paris.

[47] Nelsen R (2006). An Introduction to Copulas.

[48] Genz A, Bretz F (2009). Computation of Multivariate Normal and T Probabilities.

