# Supplementary material for: histoneHMM: Differential analysis of histone modifications with broad genomic footprints
Source: BMC Bioinformatics. 2015 Feb 22;16:60. doi: 10.1186/s12859-015-0491-6 (PMC4347972; doi:10.1186/s12859-015-0491-6)
Supplement: Additional file 1 — Supplemental information. [file 12859_2015_491_MOESM1_ESM.pdf]

Supplemental material for the article “histoneHMM: Differential analysis of histone modifications with broad genomic footprints”

Parameter estimation using gene expression data

Since both histone modifications H3K27me3 and H3K9me3 are associated with gene silencing, high occupancy values can be associated with lowly expressed genes and the low occupancy values with highly expressed genes. This way, if expression data are available, we can use this biological information, and fit one component of the mixture distribution using read counts overlapping the 20% most highly expressed genes and one component using read counts overlapping the 20% most lowly expressed genes. The 20% threshold was chosen because it clearly separated the bimodal distribution of RNA-seq expression values of very lowly expressed from genes with higher expression levels (data not shown). For the differential analysis we first estimated the parameters of each marginal distribution as outlined in the main text. Then we called modified and unmodified regions in each sample separately and used high confidence differential regions ($P(\text{modified}_x|O) > 0.95 \wedge P(\text{modified}_y|O) < 0.05 \vee P(\text{modified}_x|O) < 0.05 \wedge P(\text{modified}_y|O) > 0.95$) to estimate the covariance matrix Σ of the copula. We used the same procedures described in the main text in order to evaluate the performance of the HMM fitted with gene expression data. Supplemental Figure 1 shows that histoneHMM fitted with gene expression data yields a more significant overlap with differentially expressed genes. Supplemental Figure 2 shows that including gene expression data also improved the single sample analysis.

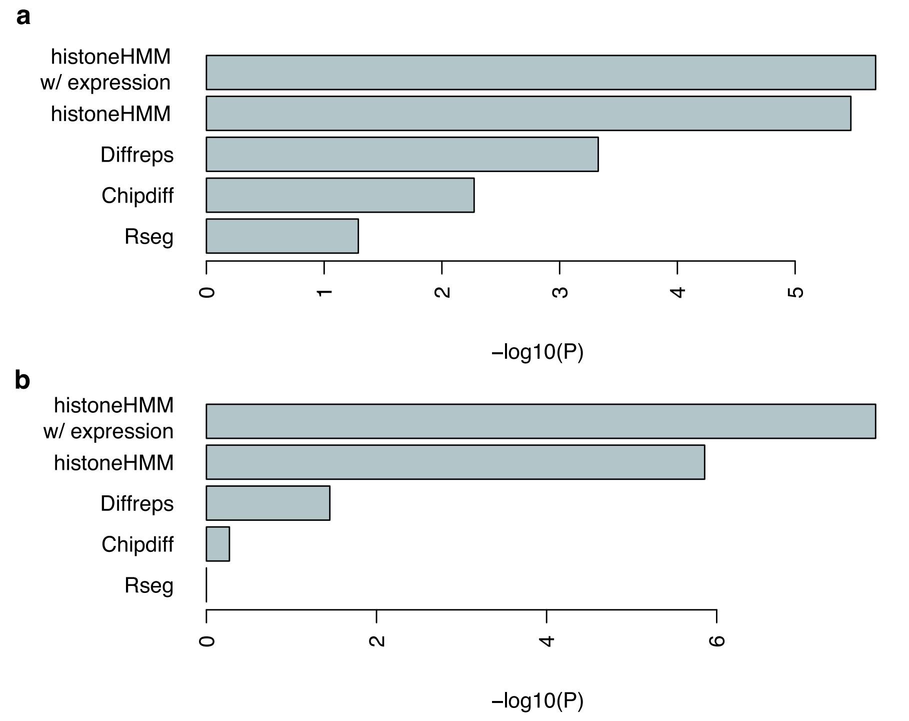

Supplemental Figure 1 Test for overlap with differentially expressed genes. a-b) Barplots of $-log_{10}(P)$ of Fisher's exact test for overlap with differentially modified genes for each of the methods. **a)** shows results for H3K27me3 in the rat strain comparison. **b)** shows results for H3K9me3 in the comparison of female and male mice.

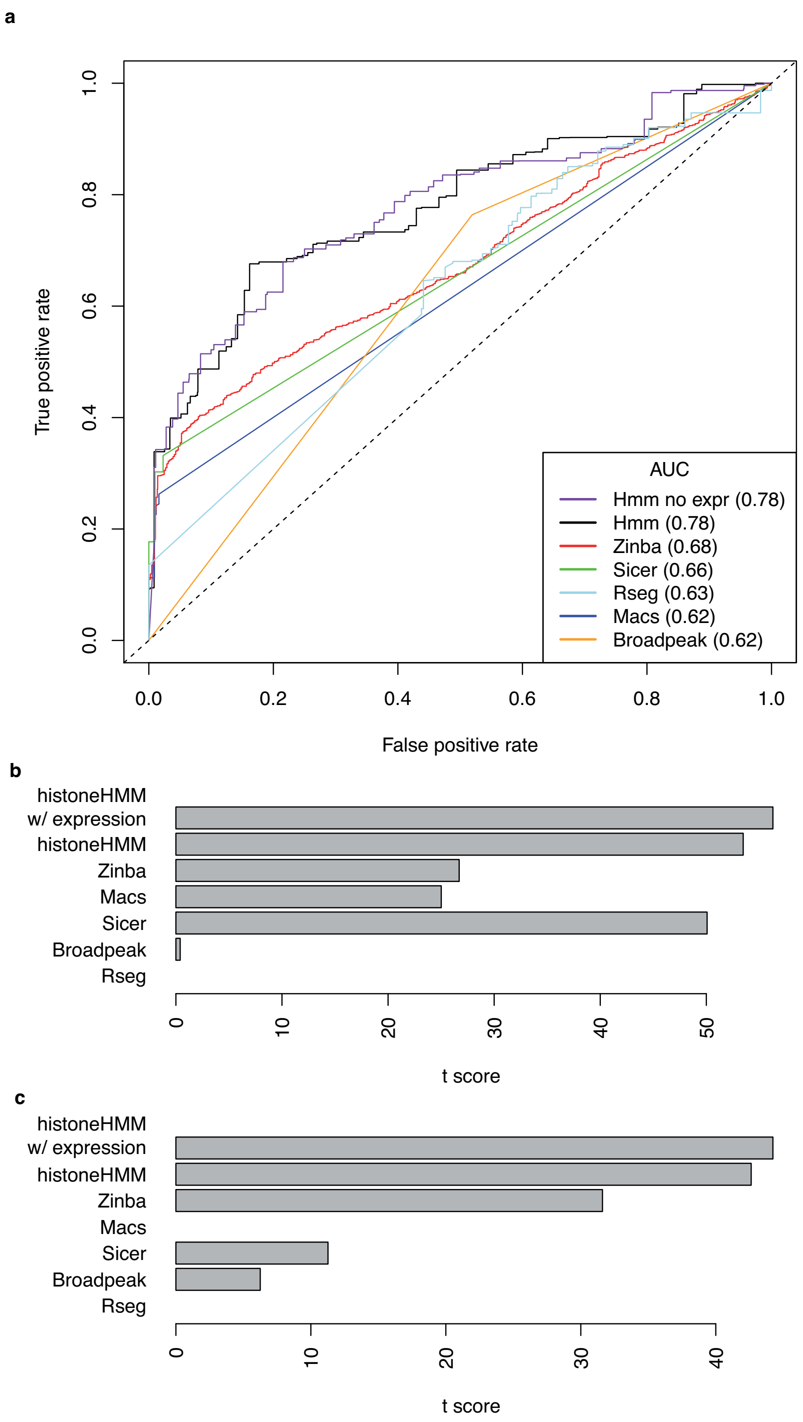

Supplemental Figure 2 Single ChIP-seq sample analysis. a) Receiver operator characteristics curves show the sensitivity and specificity of different methods for H3K27me3 peak calls evaluated using qPCR validated regions. The association of region calls with gene expression is quantified using the t-statistic for H3K27me3 **b)** and H3K9me3 **c)**.

Simulation study

We conducted an extensive simulation study to compare the performance of differential analysis methods in the absence of a large scale benchmark data set. We used our bivariate HMM to obtain parameter estimates from ChIP-seq data sets of two rat strains (see methods). Using the parameters learned from real data, we simulated data from the HMM by first sampling a hidden state path from the transition matrix and then obtaining pairs of read counts by sampling from the emission distribution of each state. To obtain a sample from the copula distribution we first sampled z-values from the underlying bivariate normal distribution and then transformed the z-values to counts using the transformation $x = F_x^{-1}(\Phi(z_x))$, where F_x^{-1} is the inverse of the marginal CDF and Φ is the CDF of the standard normal distribution. For Chipdiff and Rseg we transformed the read counts to read tag positions by uniformly sampling random start positions within each bin.

From the simulated data we reestimated the parameters of the HMM. When no gene expression information is used for training, we simply run the EM-algorithm on the simulated data. To mimmick the situation where gene expression data can be used to fit the parameters, we first determined the number of bins that belong to the genes in the top and bottom expression quintile. We randomly chose the same number of bins of the corresponding state to reestimate the emission probabilities. We evaluated the performance of the models using the receiver operator characteristic, sensitivity and specificity. Each bin was considered a data point and was labeled 1 if it was differentially modified and 0 if the hidden state was not differentialy modified.

In particular we were interested in the performance of the bivariate HMM and how it compares with simpler methods based on the univariate HMM analysis. The simplest way of calling differential regions between two samples is to compute the univariate posterior probabilities for each sample separately, apply the threshold λ to call a bin modified or not, and then compare the univariate calls with each other. The main drawback of this approach is that regions that have posterior probabilities close to the threshold can lead to differential calls although the posteriors might be almost identical. The second method circumvents this problem by computing the difference of the posterior probabilities and applying the threshold to the absolute value of it. We observed that region calls using the first method were of very low quality (data not shown). Supplemental Figure 3 shows that the bivariate HMM outperforms the univariate approach where a threshold on the absolute difference of the posterior probabilities was applied. Using the bivariate model for differential region calling, we found that the cutoff $\lambda = 0.5$ yields a sensitivity of 0.99 and a specificity of 0.96. For comparison we also simulate data from the univariate model for region calling and found that the cutoff $\lambda = 0.5$ yields a sensitivity of 0.99 and a specificity of 0.97.

We also used the simulated data to compare the performance of the bivariate HMM with the previously published methods for differential analysis: Chipdiff , Diffreps and Rseg Chipdiff is also based on a HMM and requires the user to set a minimal fold-change f between the two signals. The emissions are modeled by a binomial distribution, where the parameter p for one sample is constrained to be

at least f times greater than for the other sample. Since the exact values of both p are not available, Chipdiff integrates over a prior distribution. Diffreps partitions the genome into bins and sequentially tests for differential counts, so we used the resulting P-values in our comparison. Rseg uses a three state HMM where emissions are modeled by the distribution of the difference of two independent random variables that each follow the negative binomial distribution. The receiver operator characteristic in Supplemental Figure 3a shows that the bivariate HMM outperforms all competing methods. Interestingly the performance of the difference of two independent univariate HMMs and Rseg is almost identical. Both models treat the two samples as independent, in contrast the bivariate HMM takes the correlation between the two samples into account, and thus yields better performance. Here we used Chipdiff with a fold-change $f = 2$, however we also evaluated Chipdiff with varying f but the performance was not improved.

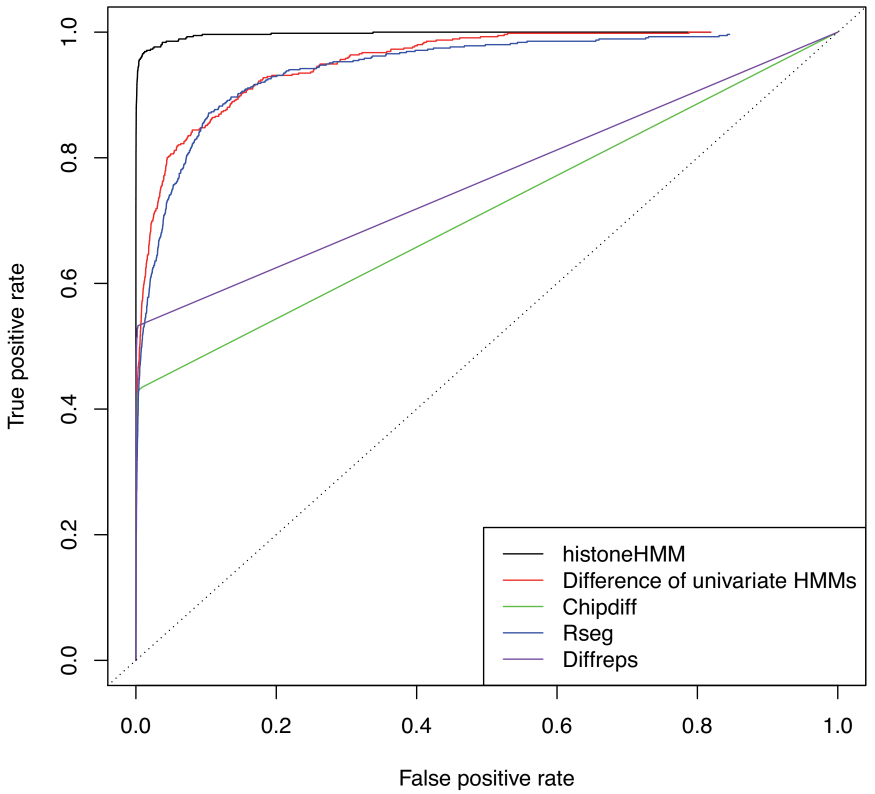

Supplemental Figure 3 Evaluation of the bivariate HMM for differential region calling. Simulation results for the identification of differentially modified regions. Performance is shown as receiver operator characteristics (ROC) for varying cutoffs of the posterior probability of the differential states in histoneHMM, the absolute difference of posterior probabilities of two univariate HMMs, the posterior probabilities for differential states of the Chipdiff and Rseg HMMs and the P-values of the Diffreps method.

Evaluation of differential calls with optimized thresholds

The evaluation of differential region calls presented in the main text is based on default significance thresholds for each method. The methods can be classified into probabilistic models that output state probabilities and hypoth-

esis testing methods that output P-values. Therefore the results might not be directly comparable. To rule out that the results presented in the main text are the consequence of the arbitrary choice of the significance thresholds, we systematically evaluated the performance with significance thresholds $x \in \{10^{-6}, 10^{-5}, 10^{-4}, 10^{-3}, 0.01, 0.02, \ldots, 0.89, 0.90\}$ and probability thresholds $1 - x$. We classified all expressed genes into differentially expressed and differentially modified regions and applied Fisher's exact test. To avoid infinite odds ratios we added a pseudo count of 1 to each cell of the contingency table. Supplemental Figure 4 shows the $-\log_{10}(P)$ of the most significant overlap for each method. For the marks H3K36me3 and H3K79me2, which are directly related to transcription, we observed such highly significant overlaps that the P-values were numerically zero, so we set them to the smallest value that can be represented numerically as a float with double precision.

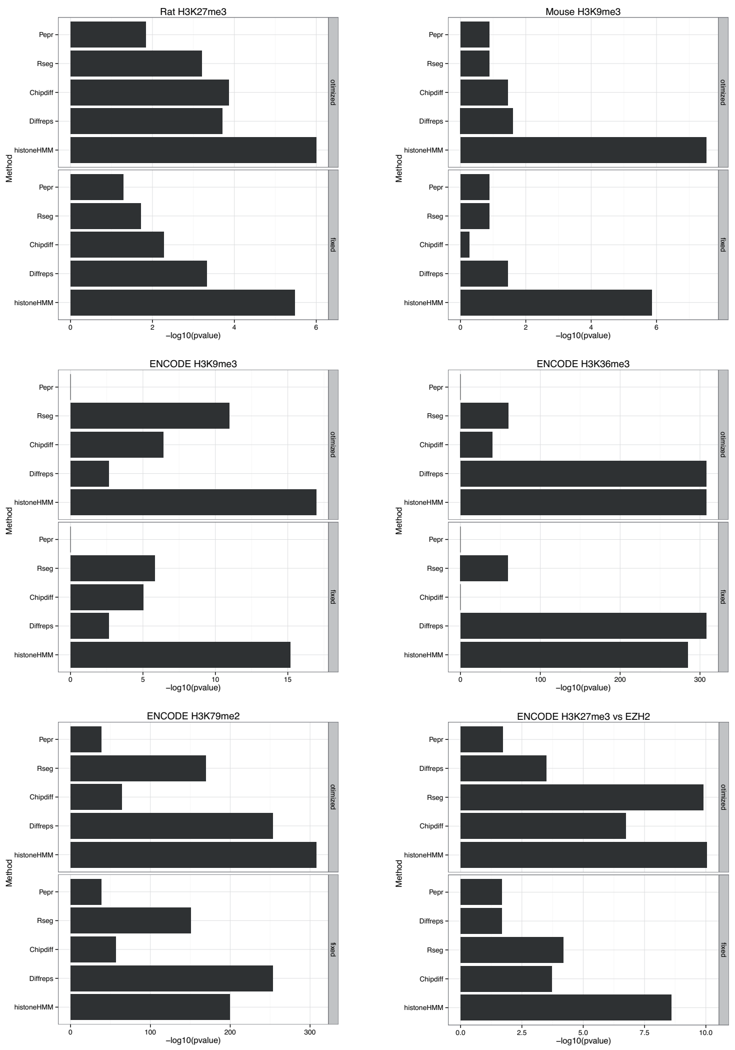

Supplemental Figure 4 Evaluation of differential region calling with optimized thresholds. The label 'fixed' refers to the thresholds used in the main text and 'optimized' refers to the thresholds that were determined through a systematic search.
